# Health effects and cost-effectiveness of a multilevel physical activity intervention in low-income older adults; results from the PEP4PA cluster randomized controlled trial

**DOI:** 10.1186/s12966-022-01309-w

**Published:** 2022-06-27

**Authors:** Katie Crist, Kelsie M. Full, Sarah Linke, Fatima Tuz-Zahra, Khalisa Bolling, Brittany Lewars, Chenyu Liu, Yuyan Shi, Dori Rosenberg, Marta Jankowska, Tarik Benmarhnia, Loki Natarajan

**Affiliations:** 1 Department of Urban Studies & Planning, UC San Diego, 9500 Gilman Drive, La Jolla, CA 92093 USA; 2 Division of Epidemiology and Community Health, School of Public Health, University of Minnesota, 1300 S 2nd Street, Minneapolis, MN 55415 USA; 3 Herbert Wertheim School of Public Health and Human Longevity Science, UC San Diego, 9500 Gilman Drive, La Jolla, CA 92093 USA; 4 Kaiser Permanente Washington Health Research Institute, 1730 Minor Avenue, Seattle, WA 98101 USA; 5 Population Sciences, Beckman Research Institute, City of Hope, 1500 E Duarte Rd, Duarte, CA 91010 USA; 6 Scripps Institution of Oceanography, UC San Diego, 9500 Gilman Drive, La Jolla, CA 92093 USA

**Keywords:** Intervention, Physical activity, Accelerometer, Older adults, Quality of life, Walking, Sensors, Community, Health coaching

## Abstract

**Background:**

Older adults are the least active population in the U.S. Low-income communities have fewer physical activity (PA) resources, contributing to less PA and increased chronic disease risk. This study assessed the effect of the multilevel, peer-led, Peer Empowerment Program 4 Physical Activity (PEP4PA) on moderate-to-vigorous PA (MVPA) and health outcomes, over 2 years of follow up.

**Methods:**

In a cluster-randomized controlled trial, 12 senior or community centers serving low-income older adults were assigned to a PA intervention (*n* = 6) or usual programming (*n* = 6) condition. PEP4PA included self-monitoring, health coaching, group walks, social support, and community advocacy to improve walking conditions. The primary outcome was daily minutes of MVPA (7-day accelerometer). Secondary outcomes included Perceived Quality of Life (PQoL), 6-Minute Walk Test (6-MWT), blood pressure (BP), and depressive symptoms at baseline, 6, 12, 18 and 24 months. Mixed effects regression models estimated the effects on outcomes between groups over time and included random effects for repeated measures and center clustering. Effect modification by sex and income status was assessed. We calculated the incremental cost per daily minute of MVPA gained in the intervention group relative to the control group to assess cost effectiveness.

**Results:**

We enrolled 476 older adults (50 + years). Participants were on average 71 years old, 76% female, 60% low income, and 38% identified as racial or ethnic minorities. Compared to the control group, intervention participants sustained roughly a 10 min/day increase in MVPA from baseline at all time points and increased mean PQoL scores from unsatisfied at baseline to satisfied at 12, 18 and 24 months. Males and higher-income groups had greater improvements in MVPA. No significant effects were observed for 6-MWT or depressive symptoms, and BP results were mixed. The incremental cost per minute MVPA gained per person was $0.25, $0.09, $0.06, and $0.05 at 6, 12, 18 and 24 months, respectively.

**Conclusions:**

PEP4PA achieved increases in MVPA and PQoL in low-income older adults, over 2 years of follow up. The peer-led, community-based intervention provides a sustainable and cost-effective model to improve health behaviors in underserved, aging populations.

**Trial registration:**

ClinicalTrials.gov (NCT02405325) March 20, 2015.

**Supplementary Information:**

The online version contains supplementary material available at 10.1186/s12966-022-01309-w.

## Background

Physical activity (PA) levels of U.S. adults over the age of 50 are lower than all other segments of the population [1, 2], and fewer than half meet PA recommendations when measured with accelerometer devices [3]. Physically inactive older adults are more likely to suffer from falls and cardiovascular diseases, cancer, obesity, functional limitations, diabetes, depression, and cognitive disorders, including Alzheimer’s disease [4–8]. Within the older adult population, PA levels are even lower among racial and ethnic minority groups and those with lower income [9, 10], which contributes to health inequities in chronic disease risk [11–14]. Low-income and high minority neighborhoods are known to have less supportive environments, such as fewer and lower quality parks and recreation centers, contributing to PA disparities [15–19]. Walk programming is often absent from senior centers despite being the preferred and perhaps simplest activity to adopt among older adults [20–23]. As in other age groups, female older adults have lower PA levels relative to males and also perceive environments as less conducive to PA [1, 3, 24]. Given that older adults will comprise 22% of the world’s population by 2050 [25], increasing older adults’ access to programs addressing known PA disparities should be a high priority [26].

The Social Ecological Model provides a multilevel framework for addressing behavior change at different levels of influence, from the individual to policy [27]. Very few PA programs address each level, which limits their ability to effect meaningful and sustainable change in the community. Empowerment theories create a mechanism for community participation and capacity building [28, 29]. In older adults especially, empowerment strategies can lead to increased agency for engaging in PA and, both directly and indirectly, to improvements in quality-of-life and depressive symptoms [8, 30–32]. Yet very few PA programs employ older adults to help deliver programs in community settings despite evidence that peer-led programs have been as successful as those led by professionals and could improve long-term sustainability and maintenance [33–36].

Peer-led, multilevel programs could also provide a more cost-effective intervention in low-income communities. It is estimated that physical inactivity is associated with roughly 11% of total healthcare spending [37]. Older adults are expected to comprise nearly a quarter of the U.S. population by 2060 and currently account for more than 1/3^rd^of all healthcare expenditures [38, 39], yet studies assessing cost-effectiveness of older adult PA interventions are scarce [40].

The purpose of this study was to examine the efficacy of the PEP4PA (Peer Empowerment Program 4 Physical Activity) intervention to improve moderate-to-vigorous PA (MVPA) and secondary health outcomes at 6, 12, 18, and 24 months in low-income community centers serving older adults. For our primary aim, we hypothesized participants in centers randomized to the PEP4PA intervention would significantly increase daily MVPA minutes (measured by accelerometry) to a greater extent than older adults in centers randomized to usual care. We hypothesized intervention participants would significantly improve secondary outcomes, including perceived quality-of-life scores (PQoL) and physical functioning (measured objectively by the 6-Minute Walk Test (6-MWT)), while decreasing their systolic and diastolic blood pressure (BP) and depressive symptoms to a greater extent than older adults in control centers. We further explored whether the program had differential effects on PA across income and sex strata. Lastly, we assessed the cost-effectiveness of PEP4PA in terms of incremental costs per MVPA minute gained, compared to usual programming in the control centers, at 6, 12, 18, and 24 months.

## Methods

### Study design and participants

The PEP4PA study was a 2-year, cluster randomized controlled trial in senior or community centers in San Diego County, California, USA that built upon a successful multilevel intervention previously delivered in retirement communities [41]. We used an effectiveness-implementation hybrid type II trial design to assess PA and health outcomes (the focus of this paper) as well as intervention implementation (future analyses). The PEP4PA study rationale, sample size calculation and protocol have been previously described in full [42]. The CONSORT and Template for Intervention Description and Replication (TIDieR) checklists are used in the reporting of this study and are provided in Additional files 1 and 2, respectively.

Twelve centers were randomized to the intervention (*n* = 6), or control (*n* = 6) condition and study measures were collected at baseline, 6, 12, 18 and 24 months. Potential centers agreed to either study condition and signed a Memorandum of Understanding (MOU) prior to randomization. Eligible senior and community centers were those primarily serving low-income populations in San Diego County, identified by having a median household income of the surrounding census tracts below 80% of the Area Median Income (AMI) in 2015, or $64,800 USD. To be eligible, all centers had to offer at least 1 physical activity (PA) class, agree to recruit participants and peer health coaches (PHCs), provide staff support for the program, have a space to accommodate group meetings and agree to the 2-year study period. Senior or community centers randomized to the usual care control condition did not receive an intervention; however, to aid in participant retention, five wellness presentations unrelated to PA were provided in conjunction with the 5 study assessments. A permuted block design was used to allocate an equal number of centers to the intervention and control conditions using a random number generator to populate the blocks. We enrolled one intervention and one control site together during each wave of recruitment to account for seasonal variation. The study statistician who oversaw randomization remained blinded to the study group assignments.

Participant recruitment, screening and informed consent occurred after centers were randomized. Participants were recruited via community mailers, presentations from study staff, flyers, information tables, community outreach, and word of mouth. Eligible participants were 50 years and over and able to walk without human assistance. Full eligibility criteria were described previously [42]. The study was approved by UC San Diego’s Institutional Review Board (Protocol # 150336) and was prospectively registered with ClinicalTrials.gov (ID # NCT02405325) prior to center enrollment. Data were collected between 2015 and 2020.

### Intervention

The PEP4PA intervention combined components of Empowerment Theory [43] and behavior change strategies from the Social Cognitive Theory [44]. Utilizing the Social Ecological Model [27] as a framework, the multilevel PEP4PA intervention employed behavior change strategies at the environmental, organizational, interpersonal, and individual level [42].

In brief, PEP4PA was designed with sustainability in mind, therefore the intervention was delivered by trained volunteer PHCs with support from senior center and research study staff. Senior center staff helped identify peer leaders, who were then trained and certified to deliver the intervention by the UC San Diego health educator. Senior center staff were responsible for administrative activities, including the provision of a group meeting space or indoor walking space when needed, advertising the program, and supporting the PHCs.

The trained PHCs led group walks twice a week, reviewed step goal progress and barriers with participants, and organized activities and events to maintain motivation. They were responsible for communicating educational tips and leading group discussions designed to provide social support, share successes and benefits, address walking challenges and identify strategies to overcome barriers. UC San Diego research staff met with PHCs weekly for the first 3 months, bi-weekly in months 3–6 and then monthly thereafter to provide support. PHCs were paid $100 USD per month for their role and intervention sites received a total of $2,200 each year for space, staffing, and funds to support the walking groups. To assess sustainability of the program, the 6 intervention centers underwent a second randomization at the 18-month time point. Three centers continued to receive financial support for the PHCs and monthly meetings with research staff and three centers had no further support or meetings for the final 6 months of the trial.

PEP4PA participants were guided in goal setting, self-monitoring, and additional effective SCT behavior change strategies [45] as they worked toward meeting individual step goals. The overall focus of the PEP4A intervention was ‘Every Step Counts’, therefore any intensity of PA was encouraged. Participants were provided with pedometers and tracked daily steps in logs they shared with their PHC on a weekly basis. All participants, regardless of baseline steps, were encouraged to gradually work toward an increase of at least 2,000 steps per day from their individual baseline, and then focus on maintaining that increase.

### Outcome measurement

Daily minutes of MVPA were assessed with a GT3X + ActiGraph accelerometer device (ActiGraph; Pensacola, FL). Participants were asked to wear the accelerometer on a hip belt for at least 10 h a day for 7 consecutive days. Raw triaxial accelerometer data were collected at 30 Hertz and compressed to 60 s epoch files using the low frequency extension. Device wear time was determined using the validated Choi algorithm [46] and days with ≥ 10 h of wear were considered valid. All participants were included in the analysis, however outcome data at each assessment were only included for participants with ≥ 4 valid days. Daily estimates of MVPA were defined using the validated Freedson cut point of 1952 counts per minute (cpm) on the vertical axis [47]. Accelerometers also provided average steps per day, which were used in sensitivity analyses.

For secondary outcomes, PQoL was assessed with the validated Perceived Quality of Life Scale (PQoL-20) [48]. Per scoring protocols, a 19-item mean was calculated with higher scores representing greater P-QoL and scores under or over 7.5 indicating dissatisfaction or satisfaction [49]. Physical functioning was assessed by the 6-MWT [50–52]. Study staff recorded the number of full and partial laps that participants completed while walking quickly for a 6-min testing period on a 20-m course. Systolic and diastolic BP (mm/Hg) were measured using an Omron HEM-705 CP cuff (Omron Healthcare, Inc., Lake Forest, IL) after participants rested for 5 min. Study staff completed three readings with ~ 2 min rest between readings (a fourth measure was taken if deemed necessary). The three closest readings were averaged. Depressive symptoms were assessed with the Center for Epidemiologic Studies Depression Scale short form (CESD-10) which has been validated in older adults [53, 54]. Greater CES-D scores indicate a higher presence of depressive symptoms.

### Intervention costs

Costs for the intervention group were estimated from a payer perspective, including all direct costs required to deliver the intervention in a community setting. Specifically, costs included personnel (e.g., PHC stipends, UC San Diego health educator trainings and check in meetings, coordination, material prep), tracking (e.g., PHC website and data storage), materials (e.g., pedometers, step logs, tablets, etc.), and overhead (funds provided to centers to support the walking groups). Costs solely for research purposes, such as personnel and participant incentives for outcome assessments, were not included. Control group costs included expenses for the wellness presentations delivered to control sites at the measurement assessments. Cumulative costs for the 0–6, 0–12, 0–18, and 0–24 month periods were calculated.

### Covariates

Covariates that were imbalanced between conditions at baseline or were known to be related to MVPA were included in outcome models. Participant demographics were collected through the baseline survey, including self-reported age, sex, race, household income, and highest education level attained. We created binary race (minority/non-minority), education (above/below college degree), and income (above/below 80% AMI) variables. An additional measure of physical function was assessed objectively at baseline using the Short Physical Performance Battery (SPPB) test, which includes a series of repeated chair stands, balance tests and a measured walk [55, 56]. Participants were asked to bring all medications to the measurement visit and medication names, dosage, and frequency were recorded by study staff. The study physician determined those taken to control BP and a binary BP medication variable was included in BP outcome models only.

### Adverse events

Initially, we recorded and assessed any adverse event reported to the research study staff or PHCs. However, since we only interacted with control participants at measurement time points, as opposed to the regular interaction of staff and PHCs with intervention participants, we began collecting adverse event information systematically from both groups at all measurement events about halfway through the study. All adverse events were reviewed by a study safety officer, a licensed physician, who determined whether they were considered a Serious Adverse Event (SAE) [57].

### Statistical analysis

Analyses were completed using Stata 16.1 (StataCorp, College Station, TX) on participant data only (i.e., PHCs were not included), while accounting for the clustered study design. To compare baseline participant characteristics between groups, we used independent group t-tests or Wilcoxon rank-sum tests for normally and non-normally distributed continuous variables, respectively, and chi-square tests for categorical variables. We conducted an intent-to-treat analysis. Mixed effects regression models assessed the intervention effect over time, with random intercepts included in all models for repeated measures within participants and clustering of participants within centers. An advantage of this modeling paradigm is that partial records can be included, avoiding the biases associated with complete case analyses. Mixed model analysis provides unbiased parameter estimates and valid inference under a missing at random assumption. Normal Q-Q plots revealed a skewed distribution of residuals, as is typical with MVPA outcomes. Mixed effects negative binomial models (using a count distribution) were able to account for skewness (and overdispersion) and were used to assess the effect of the intervention across time on minutes of MVPA per day (sum of minutes above the 1952 threshold). The intervention condition, study time point, and a two-way interaction (intervention condition x time) term were entered as fixed effects in unadjusted models. Multiple baseline characteristics were imbalanced between the intervention and control conditions, as is common in cluster randomized studies with few clusters [58, 59]. Thus, final models adjusted for baseline differences in age, sex, race, income, education, baseline physical function (SPPB), and baseline device wear time. We calculated and plotted the average marginal effect for each measurement time point by condition, to visualize the change in mean MVPA minutes by intervention group, adjusting for all covariates [60]. We tested for effect measure modification by income and sex using likelihood ratio tests comparing models with and without a 3-way interaction. Due to small cell sizes, differential effects by race or ethnicity were not conducted; however, income has been shown to be the best indicator of socioeconomic status in older adult health studies [61]. For secondary outcomes, we used mixed effects linear regression to model the intervention x time effect on PQoL, 6-MWT time, systolic and diastolic BP, and depressive symptoms; residual plots indicated that a Gaussian assumption was reasonable. Blood pressure models additionally adjusted for whether a person was taking blood pressure medication (yes/no).

We conducted sensitivity analyses to check the robustness of results. First, given the identified imbalance in baseline characteristics between groups, we employed inverse probability of treatment weighting (IPTW) using inverse propensity scores (PS) as weights to adjust for baseline differences between groups. We predicted the PS by modeling the binary outcome of intervention or control condition as a function of imbalanced baseline variables including sex, race (minority/non-minority), income (above or below 80% AMI), and baseline 6-MWT time, SPPB score, CESD-10 score, fall (yes/no) in prior year, device wear time, MVPA and a marital status (yes/no) x education (college/no college) interaction term. Balance between groups before and after weighting was evaluated using a Standardized Mean Differences (SMD) threshold of < 0.1 for each covariate included in the PS model (Additional file 3) [62]. Final IPTW models were further adjusted for age, sex, race, baseline income, education, SPPB and device wear time as covariates to adjust for any residual imbalance [63]. We additionally modeled the intervention effect on average steps per day to compare to MVPA outcomes, using negative binomial models, given the similar distribution of residuals.

### Cost-effectiveness analysis

To compute cost-effectiveness estimates, average adjusted daily minutes of MVPA gained per person in the intervention group relative to the control group was linearly interpolated from baseline to 6 months, 6 months to 12 months, 12 months to 18 months, and 18 to 24 months. Average cumulative MVPA minutes gained per person was then calculated by summing up average daily MPVA minutes gained per person during the entire course of 0–6 month, 0–12 month, 0–18 month and 0–24 month period and dividing by the number of participants. The cost-effectiveness outcome was defined as incremental cost per MVPA minute gained in the intervention group relative to the control group. It was computed by dividing the average cumulative MVPA gained per person by the difference in cumulative costs per person in intervention and control groups at 6, 12, 18, and 24 months, respectively.

## Results

**Fig. 1:**
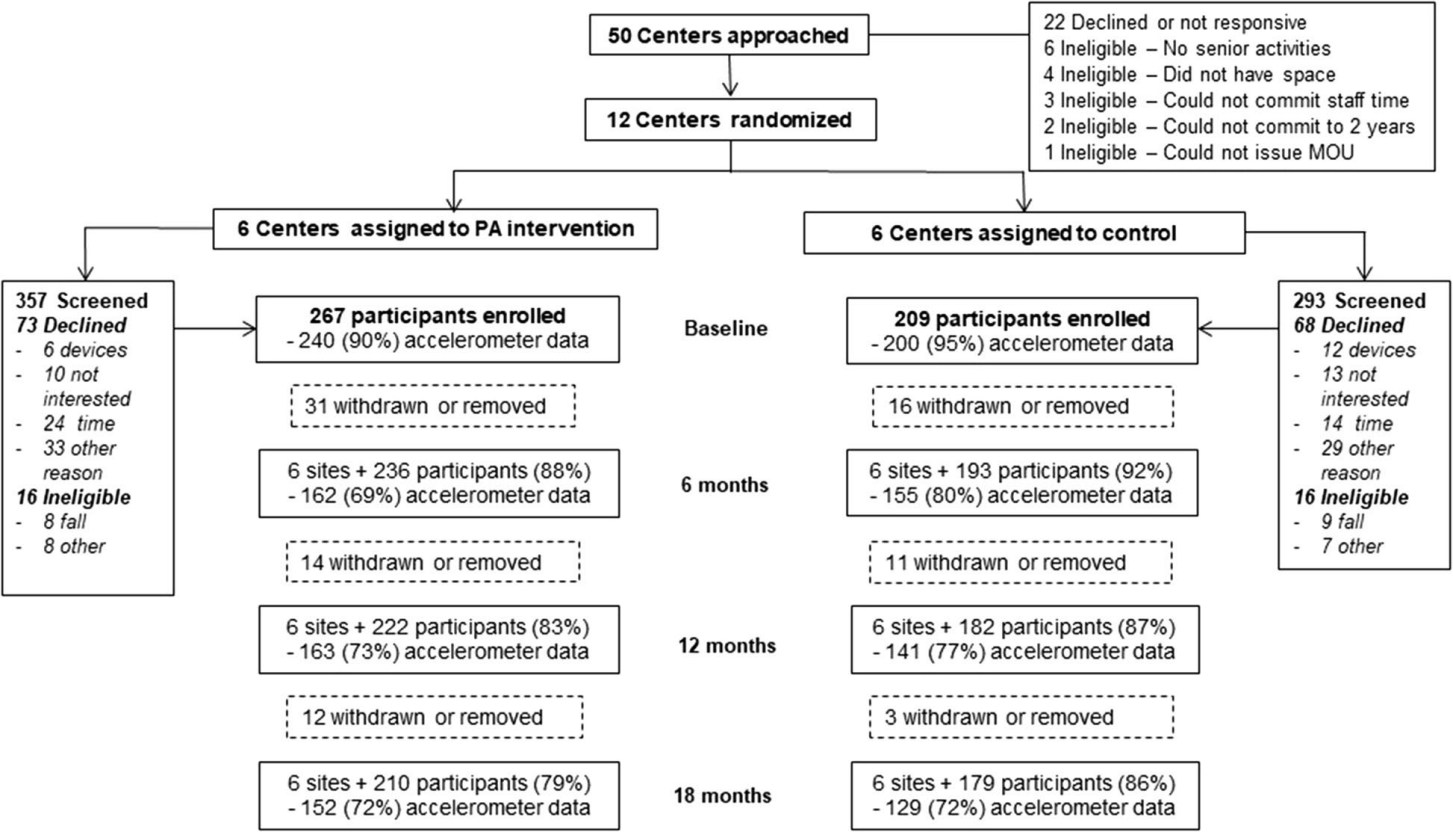
PEP4PA CONSORT diagram

We enrolled a total of 476 participants in the PEP4PA intervention and all sites completed the 2-year study (Fig. 1). There was a greater withdrawal rate in the intervention condition, with 83% and 76% of enrolled intervention participants remaining at 12 and 24-months, compared to 87% and 84% of control participants.

The mean age of PEP4PA participants at baseline was 71 years, (range 50 – 95) (Table 1). Thirty-eight percent (38%) of participants were a minority race or ethnicity, primarily Black (25%) and Hispanic (15%). The majority (60%) had an income less than 80% AMI and slightly less than half had a college education. On average, the intervention condition had a greater proportion of females and participants from minority racial/ethnic and low-income groups. At baseline, participants in the intervention condition had 10 fewer minutes of MVPA per day on average (mean 11.7 vs 22.1 min/day) and worse 6-MWT, PQoL, SPPB, and depressive symptom scores, compared to control participants. Just less than half (49%) of participants were attending the center prior to participating in the study.

**Table 1:** Baseline characteristics of the PEP4PA study sample

	**n(%) or mean(SD)**			***p*-value**
	**Overall (*N* = 476)**	**Intervention (*n* = 267)**	**Control (*n* = 209)**	
**Age (years)**	71.0 (8.9)	70.5 (8.8)	71.5 (9.1)	0.005
**Female**	359 (75.7)	215 (80.8)	144 (69.2)	0.003
**Retired or not working**	391 (86.7)	218 (89.3)	173 (83.6)	0.072
**College education or above**	211 (47.0)	85 (35.0)	126 (61.2)	0.000
**Married or living with partner**	213 (48.3)	109 (44.7)	104 (52.8)	0.090
**% Low income**	249 (59.3)	145 (65.3)	104 (52.5)	0.008
**Race**				0.000
White	297 (62.4)	120 (44.9)	177 (84.7)	
Black	117 (24.6)	113 (42.3)	4 (1.9)	
Asian	21 (4.4)	6 (2.3)	15 (7.2)	
Other	41 (8.6)	28 (10.5)	13 (6.2)	
**Hispanic ethnicity**	72 (15.2)	47 (17.7)	25 (12.0)	0.082
**Attended center prior to study**	221 (48.8)	118 (47.2)	103 (50.7)	0.454
**Baseline MVPA (min/day)**	16.1 (18.1)	11.7 (12.8)	22.1 (23.0)	0.000
**6-MWT (meters)**	392.8 (91.7)	377.4 (89.5)	417.9 (83.6)	0.000
**Perceived Quality of life**	7.6 (1.6)	7.3 (1.6)	7.9 (1.5)	0.000
**Systolic blood pressure (mm/Hg)**	129.7 (17.8)	132.2 (17.3)	130.6 (16.5)	0.321
**Diastolic blood pressure (mm/Hg)**	71.9 (10.2)	72.9 (10.8)	73.0 (10.2)	0.931
**Depressive symptoms**	6.7 (4.8)	7.1 (4.7)	5.8 (4.6)	0.000
**Fall in the previous year**	99 (21.7)	51 (20.6)	48 (23.0)	0.535

*p*-values are for independent samples t-test or chi-square test

### Main outcome

**Fig. 2:**
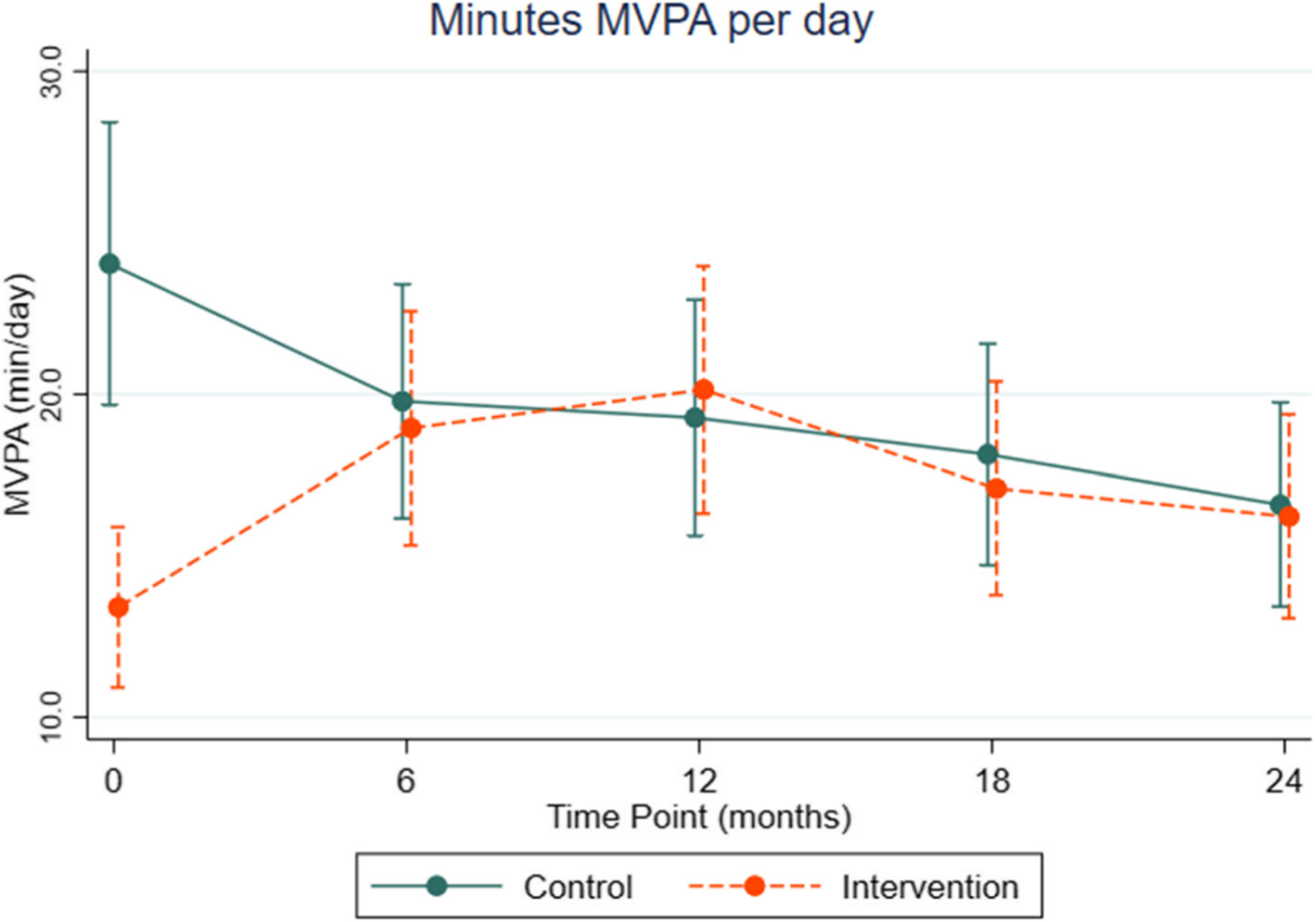
Marginal estimates and 95% confidence intervals for MVPA (min/day)

Figure 2 presents the marginal estimates of minutes of MVPA by condition across study time points from adjusted negative binomial models. Margin plot confidence intervals (CIs) indicate precision of the estimates at each time point, whereas regression coefficients and 95% CIs for the intervention condition x time interaction terms are presented in Table 2. Intervention participants significantly increased MVPA from baseline with a between group difference of roughly 10 min/day at all time points (regression coefficients in Table 2 and marginal estimates in Fig. 2). Intervention participants had the greatest gain at 12-months but maintained an average increase of 3 min of MVPA per day at 2 years from baseline, whereas control group participants decreased MVPA by 7.5 min/day across the 2-year period.

**Table 2:** Regression coefficients and 95% Confidence Intervals (CI) for MVPA (min/day) and secondary outcomes

	**6 months**			**12 months**			**18 months**			**24 months**		
	**Coef**	**95% CI**		**Coef**	**95% CI**		**Coef**	**95% CI**		**Coef**	**95% CI**	
**MVPA (min/day)**^**a**^	0.53**	**0.38**	**0.67**	0.69**	**0.54**	**0.83**	0.55**	**0.40**	**0.70**	0.54**	**0.38**	**0.70**
**PQoL score**^**b**^	0.16	-0.09	0.41	0.45**	**0.20**	**0.70**	0.35**	**0.09**	**0.61**	0.52**	**0.25**	**0.79**
**6 MWT (meters)**^**b**^	–	–	–	-5.40	-18.36	7.56	–	–	–	12.51	-1.85	26.86
**Systolic BP (mm/Hg)**^**b**^	1.18	-3.88	6.23	-4.61	-9.61	0.39	5.17*	**0.13**	**10.21**	1.96	-3.31	7.22
**Diastolic BP (mm/Hg)**^**b**^	0.16	-2.49	2.81	-0.87	-3.48	1.75	2.97*	**0.33**	**5.61**	1.73	-1.04	4.49
**CES-D score**^**b**^	0.31	-0.57	1.18	0.89	0.00	1.78	0.68	-0.23	1.60	0.84	-0.10	1.78

All models adjusted for age, gender, race, baseline income, education, baseline (SPPB), baseline device wear time. Systolic and diastolic outcome models adjusted for blood pressure medications in addition to the above variables

^*^p < 0.05

^**^p < 0.01

^a^ Negative binomial model

^b^ Mixed effects linear regression model

**Fig. 3:**
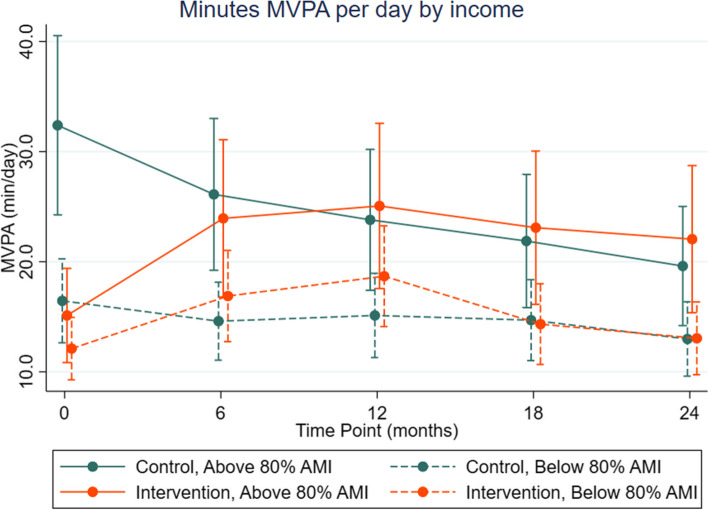
Marginal estimates and 95% confidence intervals for MVPA (min/day) for condition x time x income interaction

**Fig. 4:**
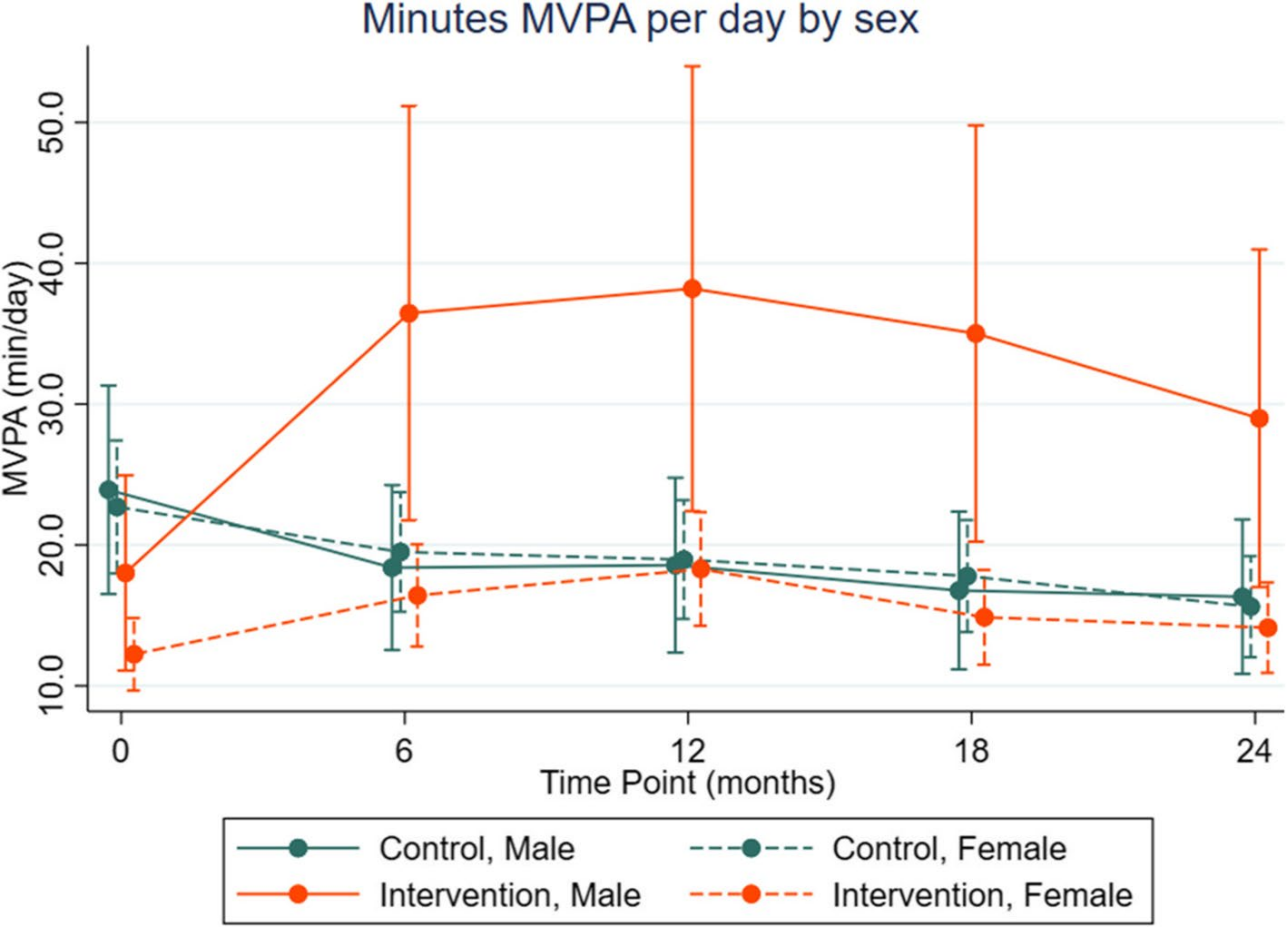
Marginal estimates and 95% confidence intervals for MVPA (min/day) for condition x time x sex interaction

The likelihood ratio test (statistic, df, p-value) comparing models with a 3-way interaction between the moderator, time point and condition were (14.61,4,0.01) for income and (12.65,4, 0.01) for sex. Based on this, we concluded that income and sex modified the intervention effect as shown in Figs. 3 and 4. Low-income participants had less MVPA than high income participants in both groups at all time points. Both low- and higher-income intervention participants increased MVPA compared to their respective control condition. Low-income intervention participants had a similar increase in MVPA as high-income intervention participants, though they had a greater decrease in MVPA from 12 to 24 months (Fig. 3).

Males in the intervention had a greater increase in MVPA from baseline and were able to sustain higher levels than females, while there was no difference in MVPA trend by sex among control participants (Fig. 4).

Results from unadjusted analyses matched adjusted models and are presented in Additional File 4.

### Secondary outcomes

**Fig. 5:**
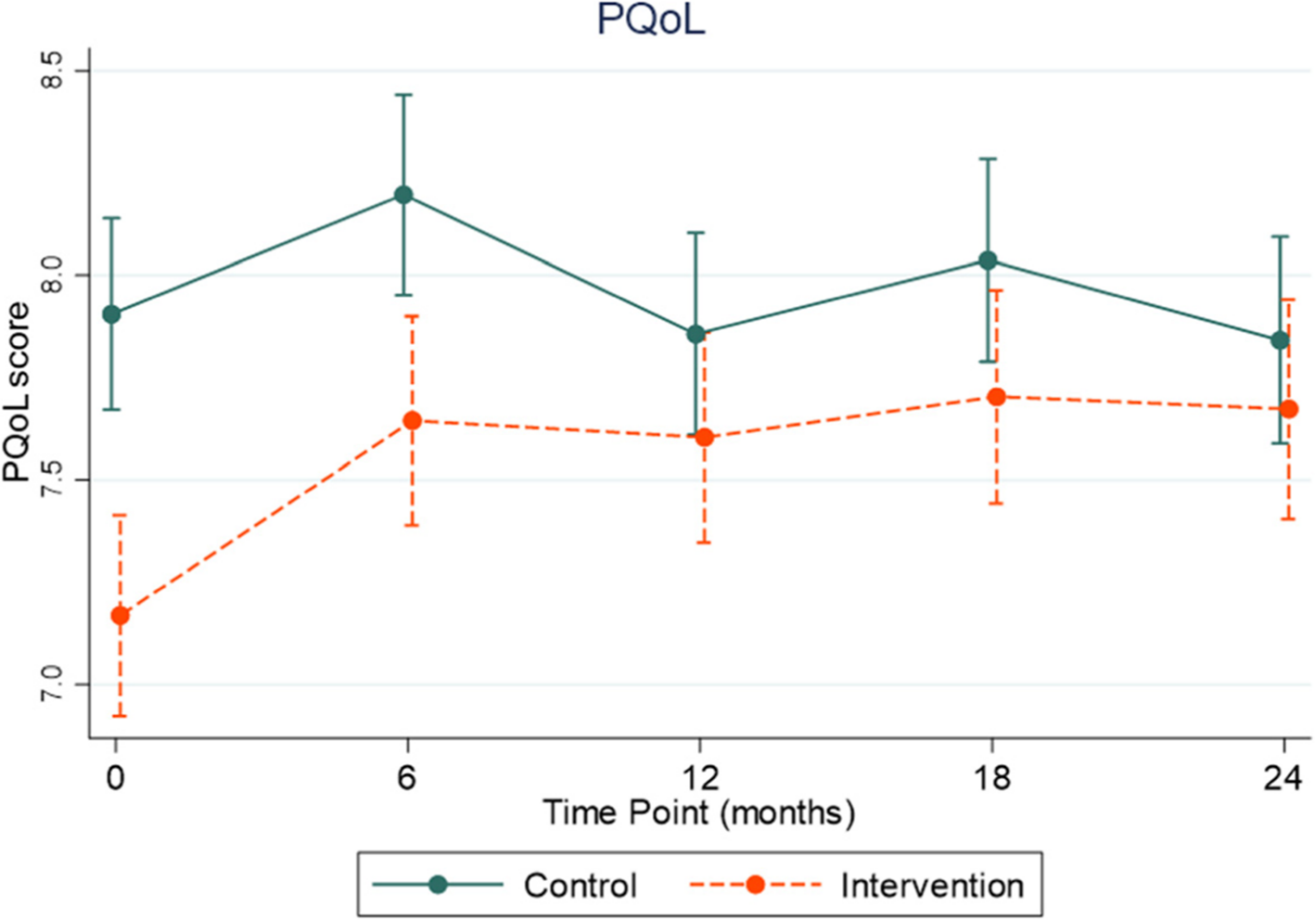
Marginal estimates and 95% confidence intervals for PQoL scores

Intervention participants had a significant increase in mean PQoL scores at 12, 18, and 24 months from baseline, compared to controls from mixed effects linear regression models (Fig. 5 and Table 2). Average marginal estimates increased from 7.2 at baseline to 7.6 or greater at all other timepoints in the intervention group.

The intervention group maintained their 6-MWT scores, while the control group had a decrease in distance walked at 24-months, though confidence intervals for the intervention effect included the null (Table 2 and Additional file 5). Control group participants experienced a greater decrease in systolic and diastolic blood pressure from baseline to 18-months, compared to intervention participants (Table 2 and Additional file 5). On average, the intervention group had higher CESD-10 scores compared to control participants, though neither group had scores that were indicative of depression (i.e. CESD-10 score ≥ 10). We did not observe an intervention effect on CESD-10 scores over time.

### Sensitivity analyses

**Fig. 6:**
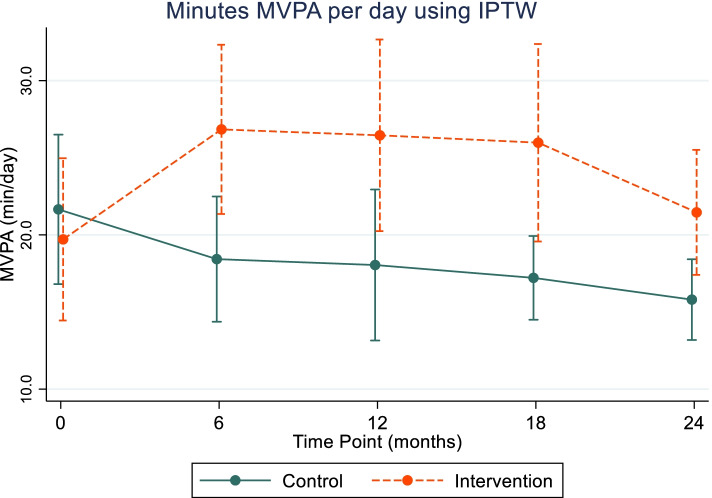
Marginal estimates and 95% confidence intervals for MVPA (min/day) using IPTW

We observed the same intervention effect on MVPA using IPTW models as non-weighted models, with intervention participants having significant increases in MVPA from baseline at all time points, compared to a decline over time in control participants (Fig. 6). Notably, we observed a decrease in systolic blood pressure of 8.7 mmHg among intervention participants from baseline to 12 months, relative to controls, in weighted models, that was not found in non-weighted models. Regression coefficients and 95% CIs for weighted models are presented in Additional file 6. Adjusted negative binomial models also showed significant increase in steps per day among intervention participants (Additional file 6).

### Adverse events

A total of 387 adverse events were reported in the intervention group, compared to 183 among control participants due to initially discrepant data collection methods, including events like muscle cramping, joint pain, respiratory issues, etc. Of those, 102 (26%) and 52 (28%) in the intervention and control groups, respectively, were considered a Serious Adverse Event (SAE) by the study’s safety officer. These included events like hospitalizations, falls and physical injuries. There were no events that were unexpected and related to study participation.

### Cost-effectiveness outcome

At 6, 12, 18, and 24 months, the cumulative per person costs of delivering the intervention were US $219, $251, $283, and $302 and US $0.48, $0.96, $1.4, and $1.9 for control participants. Costs are reported in Additional file 7. Table 3 reports the estimates of incremental costs per MVPA minute gained in the intervention group relative to the control group. The average cumulative MVPA minutes gained per person were calculated using linear interpolation of the average unadjusted daily minutes of MVPA gained, which was 9.79 min at 6 months, 11.50 min at 12 months, 9.58 min at 18 months and 10.26 at 24 months. The incremental costs per minute MVPA gained per person, obtained by dividing the difference in cumulative costs by the cumulative minutes of MVPA gained, were US$ 0.25, $0.09, $0.06, and $0.05 during 0–6, 0–12, 0–18 and 0–24-month period, respectively.

**Table 3:** Incremental costs per MVPA minute gained in intervention group vs. control group

Outcome	Intervention Group relative to Control Group			
	0–6 months	0–12 months	0–18 months	0–24 months
**Cumulative MVPA minutes gained per person (minutes)**	890.9	2838.9	4757.2	6572.6
**Difference in cumulative costs per person (US$)**	218.42	250.04	281.56	299.69
**Incremental costs per min MVPA gained per person (US$/min MVPA)**	0.25	0.09	0.06	0.05

## Discussion

PEP4PA intervention participants increased MVPA from baseline and maintained increases over the 2-year period, compared to a steady decline in MVPA among control participants. Intervention participants saw an improvement in PQoL scores from 7.2 at baseline, considered dissatisfied, to 7.6 or greater, or satisfied, at all other time points, while PQoL scores declined among control participants. Blood pressure outcomes were less clear, though weighted models accounting for baseline imbalances in individual covariates found a decrease in systolic BP in intervention participants. We observed differences by sex and income. While female and low-income participants increased MVPA, results indicated males and those with higher incomes achieved and sustained greater MVPA increases. Results are significant as even marginally more PA (e.g., 10 min per week) is associated with significant reductions in the risk and severity of chronic diseases [7, 8, 64, 65].

PA interventions of varied modalities have generally been efficacious in increasing PA in community dwelling older adults in the short-term [8, 66, 67]. However, evidence of long-term maintenance from rigorous, longitudinal studies is lacking and results are mixed [8, 66–68]. In a recent review, only studies with self-reported PA outcomes found small positive effects at 24 months, while accelerometer-measured PA outcomes were not significant [69]. Similarly, in a review of supervised PA interventions in adults 65 and over, no effects were observed beyond 6-months of follow up in any study and those with objective PA measures showed no effect at any time point [68]. In the present study, participants significantly increased device measured- MVPA, from baseline at 6,12,18, and 24 months, compared to a decline in MVPA among control participants.

Group walking interventions, specifically, have shown success increasing PA in all ages, with greater effects seen in older adults [34]. Compared to other types of PA interventions, walking shows greater PA maintenance beyond 6 months, though most prior walking interventions in older adults have only assessed outcomes up to one year and used pedometers rather than accelerometers to measure PA [34, 66]. In our previous MIPARC study (mean age 84 yrs), we found a significant, though smaller, intervention effect at multiple time points up to 1 year [41]. However, the RiAT trial, a similar peer-led intervention in retirement communities, did not observe a change in MVPA at 6 months among walkers, as assessed by activPAL devices [70]. Our study aligns with two, 12-week pedometer interventions, delivered by mail or a nurse, that found greater accelerometer-assessed MVPA after 3 and 4 years of follow up, though between group differences were less than half of what we observed at 2 years [71]. Taken together with these pedometer interventions, our findings provide evidence of the effectiveness of peer-led walking programs to increase daily PA and indicate long-term sustainment is achievable in this age group.

PA studies in older adult populations with socioeconomic diversity are scarce [8, 66, 67], and a recent review found few assessed differential effects by sex and none by income status [72]. The smaller effect observed for intervention females compared to males aligns with prior group walking studies showing larger effect sizes in interventions with both sexes than those with women only [34, 41]. Some evidence suggests men are more likely to continue to adhere to a walking program [73], and are more likely to exercise outdoors [23], which may help explain the observed difference in this study. Despite having similar motivation to be active, females report more barriers to PA, especially built environment factors affecting safety [24]. The group walking and social support components of PEP4PA may have helped overcome some of these barriers leading to increased MVPA in females, though more targeted strategies may be needed to achieve similar gains as males. Low-income intervention participants in our study had a comparable increase in MVPA as higher income participants at 12-months but did not sustain MVPA levels to the same degree from 12 to 24 months. This difference in sustainment suggests maintenance-focused programs could be directed to the lowest resource settings to support long-term health benefits. This study provides needed insight into future interventions to address PA inequalities, given the current lack of evidence from prior interventions [34, 72, 74].

A positive association between PA and health-related QoL in older adults has been established [75], though evidence of PA interventions’ ability to improve global measures of QoL is mixed. Several PA interventions assessing QoL found an improvement at time points up to 12 months [66, 76, 77], while others have not observed a difference [41, 70, 71, 78, 79]. It has been suggested that self-efficacy may mediate the relationship between PA and QoL [75, 80]. Components of the PEP4PA intervention designed to build self-efficacy, such as peer coaching, social support, group walks, and tracking PA and health improvements, may have contributed to the observed improvement in PQoL. While walking group interventions in the general adult population have improved BP, depressive symptoms, and walking tests [74, 81], the evidence is less robust for older adults. Our previous MIPARC walking study showed a decrease in BP at 6 months but not 12 months, whereas others have not found intervention effects [41, 82]. In this study, non-weighted analyses showed intervention participants’ blood pressure declined over the first 12 months then returned to baseline levels. BP in the control group did not follow a clear pattern, but there was an overall decrease across 2-years. Weighted analyses, however found a significant and clinically meaningful reduction of nearly 9 mmHg in intervention participants compared to controls at 12-months. It is possible the weighted models better accounted for unmeasured differences in BP management, driven by socioeconomic disparities between groups. Exercise programs in general have been shown to significantly reduce depressive symptoms in older adults in the short-term and in older adults experiencing depression [77, 83, 84]. Similar to our findings, other studies have not reported a change in depressive symptoms from walking interventions, even with long-term follow-up [41, 66, 70, 85]. Our finding is unsurprising given the low levels of depressive symptoms at baseline. While control participants showed a decline in 6-MWT distances over 2-years compared to intervention participants, the results were imprecise. The LIFE-P study, which focused on strength and balance activities in addition to walking in a population with lower physical functioning than the current sample, achieved an improvement in 400-m walk speeds [86]. It may be that walking alone is insufficient for improving physical function outcomes, though the trend indicates that intervention participants avoided age-related decline.

The average cumulative cost of the intervention per participant compared to controls was $250 after 1 year and $300 after 2 years of implementation. This cost is low compared to similar interventions, especially given the longer duration of the PEP4PA program [26, 87, 88]. For example, other similar programs range from an average per participant cost of $229 for the *Texercise Select* program to $2,301 for the LIFE study [89–91]. The average cost per participant in the nurse-delivered arm of the PACE-UP pedometer trial was more than $900 USD (based on 2014 exchange rate) and the cost per additional minute of MVPA was ~ $5.7 at 12 months, significantly higher than the PEP4PA intervention [92]. By comparison, the PEP4PA cost-effectiveness ratios decreased from $0.25 at 6 months to $0.05 at 24-months, far lower than other interventions, providing a financially feasible intervention.

Strengths of the study include the cluster-randomized design, the diverse, low-income population, and the device-based, long-term PA assessment. The multilevel intervention utilized a strong theoretical framework including Empowerment theory, which is a promising yet under-studied approach in older adult intervention research [32]. The peer-led intervention provides an effective strategy to improve long-term sustainability in under-resourced, community settings. The multilevel design aligns with recommendations from systematic reviews that interventions including social and environmental supports as well as a combination of behavioral and cognitive strategies are more effective in increasing PA in older adults [66–68, 78]. The center-based events combined with individual tailoring to participants’ motivations and abilities appear important for ongoing participation and larger effects [8, 66, 67, 69]. While it is likely the combination of multiple strategies is most effective [67, 68, 93, 94], the lack of conclusive evidence on strategies supporting long-term sustainability warrants further exploration of intervention components [68]. Simplifying the program to essential elements, or fine-tuning the timing of strategies, may reduce barriers to implementation and aid in wider dissemination.

There are several limitations that should be noted. Participants were recruited into a specific study condition, which could have contributed to baseline differences in individual characteristics. The cluster-randomized design was essential as the intervention involved center staff and changes to the environment around participating centers, thus cross contamination would be unavoidable with participant level randomization. However, the intervention group was less active and had more physical limitations at baseline, which is opposite of what we might expect. While we did not achieve exchangeable groups at the individual level at baseline, unadjusted and PS weighted models confirmed our main findings. There was a higher withdrawal rate in the intervention condition, though 68% of both groups had valid accelerometer data at 24-months and intervention group withdrawal rates were similar to much shorter PA interventions [66]. Participants were generally healthy, ambulatory and without serious cognitive decline, thus findings may not generalize to older adults with decreased physical and cognitive functioning. While the falls risk criteria may have excluded some participants who would have benefited from a PA program, given that the intervention encouraged unsupervised walking, this precaution was necessary to minimize falls risk.

## Conclusions

The PEP4A study provides evidence of a highly cost-effective intervention to increase PA and improve QoL in older adults over a 2-year period. The multilevel, peer-led, theory-based intervention provides a successful model for sustainable, long-term health improvements that could be implemented in other community settings for wider dissemination. It offers valuable insight for future studies to reduce persistent PA inequities by sociodemographic factors. Future work will assess implementation measures to evaluate the effectiveness and acceptability of intervention components and behavior change strategies. The considerable evidence of the benefits of regular PA, combined with low levels of PA guideline adherence and the growth of the older adult population globally, make it imperative that we identify and widely disseminate effective, sustainable, and scalable interventions to increase PA, particularly in vulnerable populations.

## Supplementary Information

**Additional file 1. **PEP4PA_CONSORT Extension for Cluster Trials 2012 Checklist.

**Additional file 2. **PEP4PA_TIDieR-Checklist-Word.

**Additional file 3. **Standardized mean differences.

**Additional file 4. **Unadjusted regression coefficients and 95% confidence intervals (CI) for all outcomes.

**Additional file 5. **Marginal estimates for secondary outcomes.

**Additional file 6. **Sensitivity analyses.

**Additional file 7. **Cumulative per person costs for intervention and control conditions.

## Data Availability

The datasets used and/or analyzed during the current study are available from the corresponding author on reasonable request.

## Abbreviations

PEP4PA: Peer Empowerment Program 4 Physical Activity
MVPA: Moderate-to-vigorous physical activity
PA: Physical Activity
MOA: Memorandum of Understanding
P-QoL: Perceived quality of life
6-MWT: Six-minute walk test
BP: Blood pressure
AMI: Area Median Income
CESD-10: Center for Epidemiologic Studies Depression Scale short form
CPM: Counts per minute
IPTW: Inverse probability of treatment weighting
PS: Propensity score
USD: U.S. Dollar
