# Supplementary material for: Health effects and cost-effectiveness of a multilevel physical activity intervention in low-income older adults; results from the PEP4PA cluster randomized controlled trial
Source: Int J Behav Nutr Phys Act. 2022 Jun 27;19:75. doi: 10.1186/s12966-022-01309-w (PMC9235144; doi:10.1186/s12966-022-01309-w)
Supplement: Supplementary file 3 — Additional file 3. Standardized mean differences. [file 12966_2022_1309_MOESM3_ESM.docx]

**Additional file 3. Standardized Mean Differences (SMD) of propensity score covariates between intervention and control groups, before and after weighting**

|  | Standardized Differences | |
| --- | --- | --- |
|  | Raw | Weighted |
|  |  |  |
| Sex | 0.327 | -0.116 |
| Race | 0.727 | 0.037 |
| Marital status x education | -0.491 | 0.104 |
| Baseline income | -0.185 | -0.056 |
| Baseline 6 MWT | -0.431 | 0.086 |
| Baseline depressive symptoms | 0.216 | 0.026 |
| Baseline fall in prior year | -0.106 | 0.037 |
| Baseline device wear time (min/day) | -0.139 | -0.091 |
| Baseline SPPB score | -0.390 | 0.035 |
| Baseline MVPA | -0.650 | -0.132 |
