# Supplementary material for: Health effects and cost-effectiveness of a multilevel physical activity intervention in low-income older adults; results from the PEP4PA cluster randomized controlled trial
Source: Int J Behav Nutr Phys Act. 2022 Jun 27;19:75. doi: 10.1186/s12966-022-01309-w (PMC9235144; doi:10.1186/s12966-022-01309-w)
Supplement: Supplementary file 4 — Additional file 4. Unadjusted regression coefficients and 95% confidence intervals (CI) for all outcomes. [file 12966_2022_1309_MOESM4_ESM.docx]

**Additional File 4.** **Unadjusted regression coefficients and 95% confidence intervals (CI) for all outcomes**

|  |  | **6 months** | | | **12 months** | | | **18 months** | | | **24 months** | | |
| --- | --- | --- | --- | --- | --- | --- | --- | --- | --- | --- | --- | --- | --- |
|  |  | **Coef.** | **95% CI** | | **Coef.** | **95% CI** | | **Coef.** | **95% CI** | | **Coef.** | **95% CI** | |
| **MVPA  (intervention x time)** |  | 0.49** | 0.37 | 0.62 | 0.67** | 0.54 | 0.80 | 0.48** | 0.34 | 0.62 | 0.51** | 0.37 | 0.65 |
| **MVPA  (Intervention x time x low income)** |  | -0.12 | -0.48 | 0.23 | -0.33 | -0.69 | 0.03 | -0.47* | -0.85 | -0.10 | -0.42* | -0.80 | -0.03 |
| **MVPA  (Intervention x time x female)** |  | -2.29* | -0.84 | -0.07 | -2.54* | -0.91 | -0.12 | -3.02** | -1.03 | -0.22 | -1.52 | -0.74 | 0.09 |
| **PQoL score** |  | 0.07 | -0.16 | 0.29 | 0.42** | 0.20 | 0.64 | 0.29* | 0.06 | 0.52 | 0.45** | 0.21 | 0.69 |
| **6 MWT (meters)** |  | -- | -- | -- | -10.33 | -22.45 | 1.79 | -- | -- | -- | 11.56 | -1.90 | 25.03 |
| **Systolic BP (mm/Hg)** |  | -0.38 | -3.86 | 3.10 | -4.35* | -7.85 | -0.86 | 3.36 | -0.31 | 7.03 | -0.94 | -4.80 | 2.91 |
| **Diastolic BP (mm/Hg)** |  | 0.06 | -1.73 | 1.85 | -1.74 | -3.54 | 0.06 | 2.21* | 0.32 | 4.10 | 0.46 | -1.53 | 2.44 |
| **CES-D score** |  | -0.02 | -0.82 | 0.77 | 0.79 | -0.02 | 1.60 | 0.46 | -0.38 | 1.29 | 0.33 | -0.53 | 1.19 |
