# Supplementary material for: Health effects and cost-effectiveness of a multilevel physical activity intervention in low-income older adults; results from the PEP4PA cluster randomized controlled trial
Source: Int J Behav Nutr Phys Act. 2022 Jun 27;19:75. doi: 10.1186/s12966-022-01309-w (PMC9235144; doi:10.1186/s12966-022-01309-w)
Supplement: Supplementary file 5 — Additional file 5. Marginal estimates for secondary outcomes. [file 12966_2022_1309_MOESM5_ESM.docx]

**Additional file 5. Marginal estimates for secondary outcome from mixed effects linear regression models**

**Figure 5.1. Marginal estimates and 95% confidence intervals for 6-MWT (meters)**

**Figure 5.2. Marginal estimates and 95% confidence intervals for systolic BP (mmHg)**

**Figure 5.3 Marginal estimates and 95% confidence intervals for diastolic BP (mmHg)**

**Figure 5.4 Marginal estimates and 95% confidence intervals for diastolic BP (mmHg)**
