# Supplementary material for: Health effects and cost-effectiveness of a multilevel physical activity intervention in low-income older adults; results from the PEP4PA cluster randomized controlled trial
Source: Int J Behav Nutr Phys Act. 2022 Jun 27;19:75. doi: 10.1186/s12966-022-01309-w (PMC9235144; doi:10.1186/s12966-022-01309-w)
Supplement: Supplementary file 6 — Additional file 6. Sensitivity analyses. [file 12966_2022_1309_MOESM6_ESM.docx]

**Additional file 6. Sensitivity analyses outcomes**

**Figure 6.1 Regression coefficients and 95% confidence intervals (CI) for all outcomes using IPTW**

| **Outcome** |  | **6 months** | | | **12 months** | | | **18 months** | | | **24 months** | | |
| --- | --- | --- | --- | --- | --- | --- | --- | --- | --- | --- | --- | --- | --- |
|  |  | **Coef.** | **95% CI** | | **Coef.** | **95% CI** | | **Coef.** | **95% CI** | | **Coef.** | **95% CI** | |
| **MVPA (Intervention x time)^a^** |  | **0.36*** | **0.01** | **0.71** | **0.51**** | **0.16** | **0.87** | **0.40*** | **0.04** | **0.77** | **0.46*** | **0.06** | **0.86** |
| **MVPA (Intervention x time x low income)^a^** |  | 0.12 | -0.50 | 0.75 | 0.20 | -0.48 | 0.88 | -0.17 | -0.83 | 0.49 | -0.25 | -0.82 | 0.32 |
| **MVPA (Intervention x time x female) ^a^** |  | -0.01 | -0.65 | 0.62 | -0.05 | -0.90 | 0.80 | -0.26 | -1.07 | 0.54 | -0.01 | -0.72 | 0.70 |
| **PQoL score** |  | 0.23 | -0.19 | 0.65 | **0.39*** | **0.05** | **0.73** | 0.38 | -0.15 | 0.91 | **0.51*** | **0.00** | **1.03** |
| **6 MWT (meters)** |  | -- | -- | -- | -6.90 | -25.02 | 11.21 | -- | -- | -- | 8.56 | -23.65 | 40.77 |
| **Systolic BP (mm/Hg)** |  | -2.93 | -12.64 | 6.77 | **-8.7**** | **-14.58** | **-2.91** | 2.95 | -3.80 | 9.69 | -1.62 | -8.02 | 4.78 |
| **Diastolic BP (mm/Hg)** |  | -0.70 | -5.06 | 3.66 | -2.26 | -5.95 | 1.43 | 3.58 | -1.98 | 9.14 | 2.26 | -1.74 | 6.26 |
| **CES-D score** |  | -0.27 | -1.16 | 0.63 | 0.52 | -0.25 | 1.28 | 0.35 | -0.53 | 1.24 | 0.43 | -0.65 | 1.51 |

^a^Negative binomial model ^b^Mixed effects linear regression model

All models adjusted for age, sex, race, baseline income, education, baseline (SPPB), baseline device wear time. Systolic and diastolic outcome models adjusted for blood pressure medications in addition to the above variables.

*p<0.05 ** p<0.01

**Figure 6.2 Regression coefficients and 95% confidence intervals (CI) for all average steps per day from negative binomial models**

| **Outcome** |  | **6 months** | | | **12 months** | | | **18 months** | | | **24 months** | | |
| --- | --- | --- | --- | --- | --- | --- | --- | --- | --- | --- | --- | --- | --- |
|  |  | **Coef.** | **95% CI** | | **Coef.** | **95% CI** | | **Coef.** | **95% CI** | | **Coef.** | **95% CI** | |
| **Step (Intervention x time)** |  | **0.25**** | **0.19** | **0.31** | **0.31**** | **0.25** | **0.37** | **0.24**** | **0.18** | **0.31** | **0.25**** | **0.19** | **0.32** |

Model adjusted for age, sex, race, income, education, baseline SPPB, baseline device wear time

*p<0.05, **p<0.01

**Figure 6.3 Marginal estimates and 95% confidence intervals for average steps per day from negative binomial model**
