# Supplementary material for: Health effects and cost-effectiveness of a multilevel physical activity intervention in low-income older adults; results from the PEP4PA cluster randomized controlled trial
Source: Int J Behav Nutr Phys Act. 2022 Jun 27;19:75. doi: 10.1186/s12966-022-01309-w (PMC9235144; doi:10.1186/s12966-022-01309-w)
Supplement: Supplementary file 7 — Additional file 7. Cumulative per person costs for intervention and control conditions. [file 12966_2022_1309_MOESM7_ESM.docx]

**Additional file 7. Cumulative per person costs for intervention and control conditions**

| **Category** | **Intervention Group** | | |  | **Control Group** | | | |
| --- | --- | --- | --- | --- | --- | --- | --- | --- |
|  | 0-6 months | 0-12 months | 0-18 months | 0-24 months | 0-6 months | 0-12 months | 0-18 months | 0-24 months |
| **Personnel, US$** | 11,117 | 16,326 | 21,608 | 25,185 | 100 | 200 | 300 | 400 |
| **Web database/software, US$** | 37,450 | 38,899 | 40,349 | 41,798 | 0 | 0 | 0 | 0 |
| **Materials, US$** | 8,779 | 9,583 | 10,307 | 10,690 | 0 | 0 | 0 | 0 |
| **Overhead, US$** | 1100 | 2200 | 3300 | 3850 | 0 | 0 | 0 | 0 |
| **Cumulative costs, US$** | 58,445 | 67,008 | 75,564 | 80,523 | 100 | 200 | 300 | 400 |
| **Average cumulative costs per person, US$** | 218.9 | 251.0 | 283.0 | 301.6 | 0.48 | 0.96 | 1.44 | 1.91 |
